# Aberrant RNA sensing in regulatory T cells causes systemic autoimmunity

**DOI:** 10.1126/sciadv.adk0820

**Published:** 2024-03-01

**Authors:** Domnica Luca, Sumin Lee, Keiji Hirota, Yasutaka Okabe, Junji Uehori, Kazushi Izawa, Anna-Lisa Lanz, Verena Schütte, Burcu Sivri, Yuta Tsukamoto, Fabian Hauck, Rayk Behrendt, Axel Roers, Takashi Fujita, Ryuta Nishikomori, Min Ae Lee-Kirsch, Hiroki Kato

**Affiliations:** ^1^ Institute of Cardiovascular Immunology, Medical Faculty, University Hospital Bonn, University of Bonn, Bonn, Germany.; ^2^ Division of Integrated Life Science, Graduate School of Biostudies, Kyoto University, Kyoto, Japan.; ^3^ Laboratory of Regulatory Information, Institute for Life and Medical Sciences, Kyoto University, Kyoto, Japan.; ^4^ Laboratory of Integrative Biological Science, Institute for Life and Medical Sciences, Kyoto University, Kyoto, Japan.; ^5^ Laboratory of Immune Homeostasis, WPI Immunology Frontier Research Center, Osaka University, Osaka, Japan.; ^6^ Center for Infectious Disease Education and Research, Osaka University, Osaka, Japan.; ^7^ Laboratory of Immunology, Institute for Frontier Life and Medical Sciences, Kyoto University, Kyoto, Japan.; ^8^ Department of Pediatrics, Kyoto University Graduate School of Medicine, Kyoto, Japan.; ^9^ Division of Pediatric Immunology and Rheumatology, Department of Pediatrics, Dr. von Hauner Children's Hospital, University Hospital, Ludwig-Maximilians-Universität München, Munich, Germany.; ^10^ Munich Centre for Rare Diseases (M-ZSE), University Hospital, Ludwig-Maximilians-Universität München, Munich, Germany.; ^11^ Institute of Clinical Chemistry and Clinical Pharmacology, University Hospital Bonn, Bonn, Germany.; ^12^ Institute of Immunology, University of Heidelberg, Heidelberg, Germany.; ^13^ Department of Pediatrics and Child Health, Kurume University School of Medicine, Kurume, Japan.; ^14^ Department of Pediatrics, University Hospital Carl Gustav Carus and Medical Faculty, Technische Universität Dresden, Dresden, Germany.; ^15^ University Center for Rare Diseases, University Hospital Carl Gustav Carus and Medical Faculty, Technische Universität Dresden, Dresden, Germany.

## Abstract

Chronic and aberrant nucleic acid sensing causes type I IFN–driven autoimmune diseases, designated type I interferonopathies. We found a significant reduction of regulatory T cells (T_regs_) in patients with type I interferonopathies caused by mutations in *ADAR1* or *IFIH1* (encoding MDA5). We analyzed the underlying mechanisms using murine models and found that T_reg_-specific deletion of *Adar1* caused peripheral T_reg_ loss and *scurfy*-like lethal autoimmune disorders. Similarly, knock-in mice with T_reg_-specific expression of an MDA5 gain-of-function mutant caused apoptosis of peripheral T_regs_ and severe autoimmunity. Moreover, the impact of ADAR1 deficiency on T_regs_ is multifaceted, involving both MDA5 and PKR sensing. Together, our results highlight the dysregulation of T_reg_ homeostasis by intrinsic aberrant RNA sensing as a potential determinant for type I interferonopathies.

## Teaser

Dysregulated regulatory T cells contribute to the onset of systemic autoimmunity in patients with type I interferonopathies.

## INTRODUCTION

Type I interferonopathies, including Aicardi-Goutières syndrome (AGS), are rare monogenic autoinflammatory diseases commonly characterized by continuous production of antiviral type I interferons (IFN-I) and a striking variety of symptoms (*1*, *2*). AGS is caused by mutations in genes that are involved in nucleic acid metabolism or sensing, including loss-of-function mutations in *TREX1*, *SAMHD1*, *RNASEH2A-C*, and *ADAR1* and gain-of-function mutations in *IFIH1*, which encodes the double-stranded RNA (dsRNA) sensor MDA5 (*3*–*7*). While the AGS-causing genes function as components of innate immune pathways, some patients with AGS develop signs of systemic lupus erythematosus (SLE), a paradigm autoimmune disease (*8*, *9*). ADAR1 catalyzes the editing of adenosine to inosine in dsRNA and thereby prevents the recognition of self-RNA by MDA5 (*10*). ADAR1 deficiency causes the aberrant production of IFN-I with up-regulation of IFN-stimulated genes (ISGs) designated IFN signature. In murine models, ADAR1 deficiency causes embryonic lethality, which is delayed by concurrent deletion of MDA5 or MAVS (*10*–*12*). Furthermore, ADAR1 deficiency activates PKR, OAS/RNase L, and ZBP1 (*13*–*16*), leading to transcriptional arrest and cell death via apoptosis or necroptosis. Naturally occurring regulatory T cells (T_regs_) express the transcriptional factor FOXP3 and are indispensable for maintaining immune tolerance (*17*). T_reg_ loss or dysfunction caused by mutations in *FOXP3* or other T_reg_ signature genes results in severe or fatal autoimmune disease in humans and mice (*18*–*23*). In mouse models of viral infection and inflammatory disease, IFN-I can diminish the immunosuppressive capacity of T_regs_ (*24*, *25*). However, the contribution of T_regs_ to the pathogenesis of type I interferonopathies remains unclear. Here, we investigated the T_reg_ population in patients with AGS carrying mutations in *ADAR1* or *IFIH1* and analyzed the changes potentially resulting in pathogenesis.

## RESULTS

### Patients with AGS have a decreased frequency of peripheral effector T_regs_

**Fig. 1.**
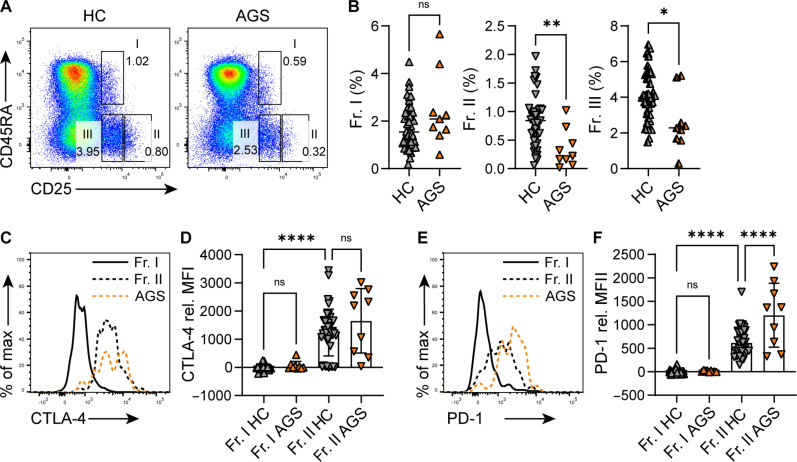
Patients with AGS caused by *ADAR1* or *IFIH1* mutations have a decreased frequency of peripheral effector T_regs_. (**A**) Representative flow cytometry (FC) plots of CD25 and CD45RA expression on CD4^+^ T cells from controls or patients with AGS. Fr. I: CD25^low^CD45RA^+^ suppressive resting T_regs_, Fr. II: CD25^hi^CD45RA^−^ highly suppressive effector T_regs_, and Fr. III: CD25^low^CD45RA^−^ (FOXP3^low^) nonsuppressive T cells. (**B**) Summarized percentages of Fr. I, Fr. II, and Fr. III from all controls and patients with AGS analyzed in this study. (**C** to **F**) Representative histograms of CTLA-4 and PD-1 expression on Fr. I (solid line) and Fr. II (dotted line) and summarized mean fluorescence intensity (MFI) values from controls (black) and patients with AGS (yellow), relative to Fr. I in healthy controls (HC). The samples from patients with AGS have been analyzed one at a time (in two instances, two at a time), together with control samples from healthy donors. The dot plots shown here contain pooled data from respective analyses. Samples from patients 1 and 4 have been analyzed at four [three for CTLA-4 and PD-1 expression; (D) and (F) and fig. S1D] and two different time points (fig. S1A), and the mean is represented as one symbol in pooled-data dot plots (B, D, and F); otherwise, each symbol represents one individual. Statistics were calculated using Student’s *t* test with Welch’s correction (B) and one-way ANOVA (D and F); *P ≤ 0.05; **P ≤ 0.01; ****P ≤ 0.0001; ns, not significant, *P* > 0.05.

To examine the T_reg_ population in peripheral blood mononuclear cells (PBMCs) from patients with *ADAR1* or *IFIH1* mutations (table S1), we gated three primary fractions out of the CD4^+^ T cell population (Fig. 1A), based on the expression level of CD25 and CD45RA: CD25^low^CD45RA^+^ suppressive resting T_regs_ (Fr. I), CD25^hi^CD45RA^−^ highly suppressive effector T_regs_ (Fr. II), and CD25^low^CD45RA^−^(FOXP3^low^) nonsuppressive T cells (Fr. III), as reported previously (*26*). We found no difference between the percentages of resting T_regs_ (Fr. I); however, the effector T_regs_ (Fr. II) that are considered the primary suppressive T_regs_ were significantly decreased in patients with AGS compared to controls (Fig. 1B). The FOXP3^low^ nonsuppressive T cells (Fr. III) were also significantly reduced (Fig. 1B). Notably, we closely analyzed the T_reg_ populations of two patients (1 and 4) at different time points and observed a continual reduction of effector T_regs_ in Fr. II (fig. S1A). Considering that all patients were children at the time of analysis, we compared PBMCs from healthy adults (aged 20 to 50 years) and children (aged 2 to 18 years) and found a similar percentage of 0.5 to 2% of effector T_regs_ in Fr. II in both groups (fig. S1B). The inhibition of Janus kinase (JAK)/signal transducer and activator of transcription (STAT) signaling, primarily by blocking JAK1 and JAK2, is a favorable treatment for some patients with type I interferonopathies (*27*–*30*). The inhibition of JAK3, which forms a dimer with JAK1 in interleukin-2 (IL-2)/IL-2R signaling, has been reported to reversibly down-regulate the expression of FOXP3 in T_regs_ (*31*). Notably, we detected no significant difference in fractions I to III as well as CD4^+^CD25^+^FOXP3^hi^ cells between untreated and JAK inhibitor–treated patients with AGS (fig. S1C).

T_regs_ express cytotoxic T lymphocyte–associated protein 4 (CTLA-4) that is crucial for their suppressive function, primarily by blocking CD80/CD86 signaling on antigen-presenting cells (*32*, *33*). Consistent with previous studies, CTLA-4 was highly expressed in effector T_regs_ and less expressed in resting T_regs_ in healthy controls (*26*), whereas there was no difference in its expression between controls and patients with AGS (Fig. 1, C and D).

Programmed cell death protein 1 (PD-1) is known to inhibit T cell antigen receptor signaling, which is required for T_reg_ functions (*34*), and its blockade has been reported to augment T_reg_ suppressive capacity (*35*–*37*). We similarly found its expression to be higher on Fr. II compared to Fr. I of healthy controls (Fig. 1, E and F). On Fr. II of effector T_regs_, PD-1 expression was significantly increased in patients with AGS compared to healthy controls (Fig. 1, E and F). Notably, in patients 1 and 4, PD-1 expression was continually increased at different time points we have analyzed (fig. S1D). This suggests a possible attenuation of effector T_reg_ suppressive activity along with the significant reduction of Fr. II in patients with AGS.

### *Adar1* deletion in T_regs_ causes T_reg_ loss and a *scurfy*-like lethal phenotype in mice

**Fig. 2.**
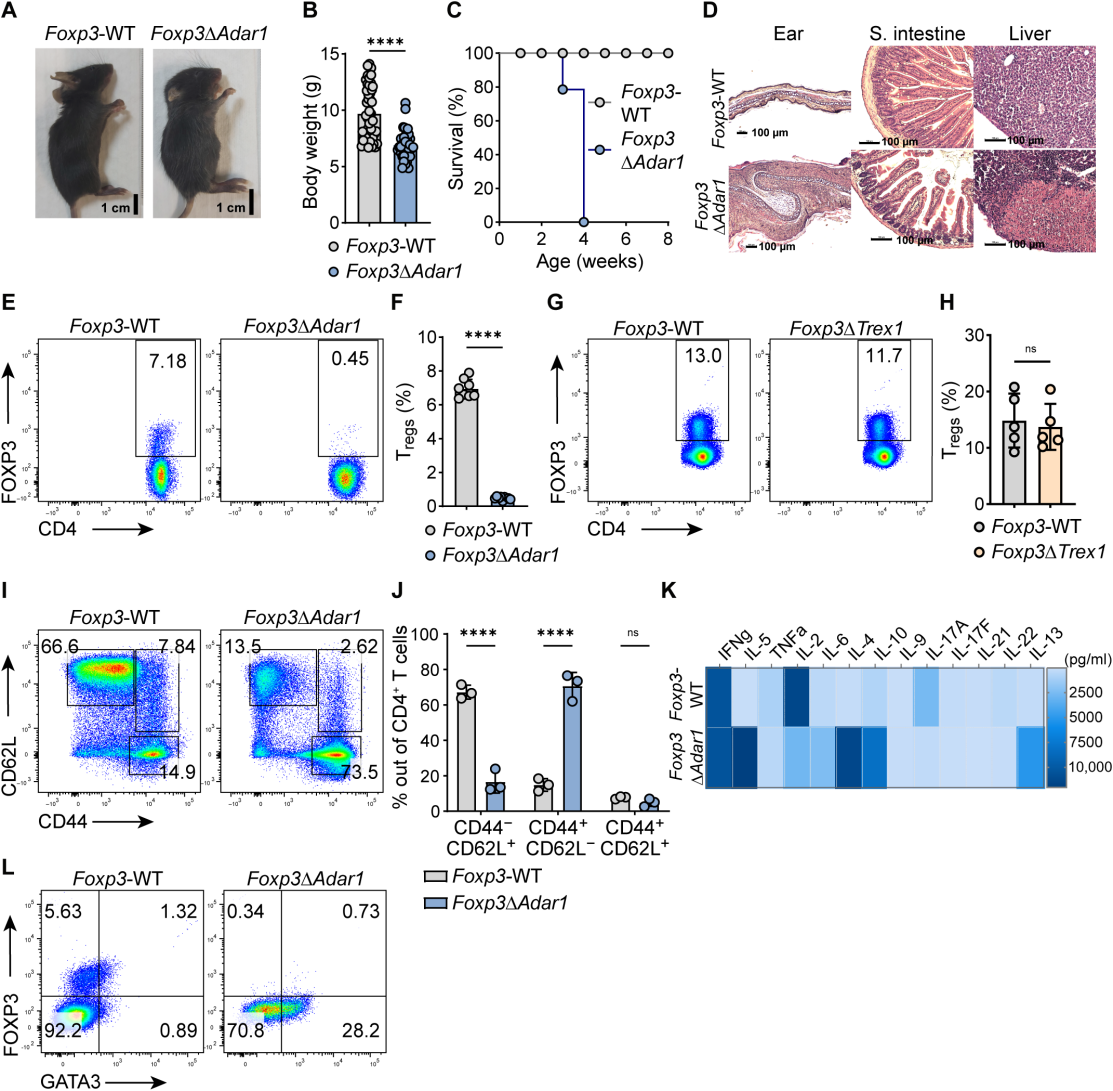
*Adar1* deletion in T_regs_ causes T_reg_ loss and a *scurfy*-like phenotype in mice. (**A** and **B**) Pictures and body weight measurements of 3-week-old *Foxp3*-WT (*n* = 44) and *Foxp3*^Δ*Adar1*^ (*n* = 36) mice. (**C**) Survival graphs of *Foxp3*-WT and *Foxp3*^Δ*Adar1*^ mice (*n* = 7, but all *Foxp3*^Δ*Adar1*^ mice developed a severe phenotype and were sacrificed at latest 4 weeks after birth). (**D**) Representative H&E staining images. S. intestine, small intestine. Scale bars, 100 μm. (**E** to **H**) Representative FC plots and summarized percentages (%) of CD4^+^FOXP3^+^ T_regs_ in the spleens. (**I** and **J**) Representative FC plots and summarized percentages of naïve (CD44^−^CD62L^+^), effector (CD44^+^CD62L^−^), and memory (CD44^+^CD62L^+^) CD4^+^ T cells in the spleens. (**K**) ELISA heatmap of indicated cytokines measured in supernatants from enriched CD4^+^ T cells (pg/ml), stimulated overnight with anti-CD3/anti-CD28 antibody–coated beads. (**L**) Representative FC plots of FOXP3^+^ versus GATA3^+^CD4^+^ T cells after overnight culture (without stimulation). Panels (E) to (J) are representative of ≥3 independent experiments with ≥3 mice per group. Panels (K) and (L) are representative of two experiments with two mice per group. In dot plots, each symbol indicates an individual mouse. Statistics were calculated using Student’s *t* test; ****P ≤ 0.0001; ns, not significant, *P* > 0.05.

The availability of samples from patients with AGS is limited; therefore, we used murine models to gain deeper mechanistic insights on the effects of ADAR1 deficiency and constitutive MDA5 signaling in T_regs_. Because systemic deletion of *Adar1* results in embryonic lethality (*11*), we aimed to assess the intrinsic effect of ADAR1 ablation in T_regs_ and generated mice with T_reg_-specific *Adar1* deletion. We intercrossed *Adar1*^fl^ mice with *Foxp3*^YFP-Cre^ mice and generated *Foxp3*^Cre/Cre^ *Adar1*^fl/fl^ female or *Foxp3*^Cre/Y^ *Adar1*^fl/fl^ male mice, herein referred to as *Foxp3*^Δ*Adar1*^. The *Foxp3*^Δ*Adar1*^ mutant mice exhibited growth retardation (Fig. 2, A and B) compared to littermate control mice—*Foxp3*^Cre/Cre^ or *Foxp3*^Cre/Y^ *Adar1*^+/+^, referred to as *Foxp3*-WT (wild type). Mutant mice died within 4 weeks of birth (Fig. 2C) and exhibited scaly skin on the tail, ears, and eyelids. By 3 weeks of age, these mice also exhibited general splenomegaly, lymphadenopathy (fig. S2, A and B), and thymic atrophy. On the basis of histologic evaluation, *Foxp3*^Δ*Adar1*^ mice exhibited severe tissue damage with immune cell infiltration in the skin dermis, liver parenchyma, lung interstitium, kidney, and intestine (Fig. 2D and fig. S2C). We also noted the up-regulation of chemokines and cytokines, including *Cxcl10*, in the spleen, liver, kidneys, and lymph nodes, along with the up-regulation of *Il-6* in the spleen and lymph nodes (fig. S2D). These phenotypes resemble those of the *scurfy* mice with *Foxp3* mutations (*33*). Considering that autoimmune symptoms in *scurfy* mice are caused by the loss of T_regs_ (*38*), we subsequently examined the T_reg_ compartment and found an almost complete depletion of FOXP3^+^ T_regs_ in the spleen and lymph nodes of 3-week-old *Foxp3*^Δ*Adar1*^ mutant mice (Fig. 2, E and F, and fig. S2, E to G). In contrast, the T_reg_-specific deletion of another AGS-related gene, *Trex1*, using the same *Foxp3*^YFP-Cre^ mice, did not change the percentage and total number of T_regs_ in the spleen (Fig. 2, G and H, and fig. S2H). We detected a larger percentage of splenic FOXP3^+^ T_regs_ in mice at an earlier stage of growth, such as 1-week-old *Foxp3*^Δ*Adar1*^ mice, compared to 3-week-old mice, albeit lower than in age-matched littermate controls (fig. S3A). Furthermore, 1-week-old *Foxp3*^Δ*Adar1*^ mice exhibited intact thymuses, and we found no differences in the percentages of thymic FOXP3^+^ T_regs_ as well as CD4^+^, CD8^+^, and CD4^+^CD8^+^ T cells (fig. S3, A and B). These data suggest that ADAR1 deficiency did not impair thymic T_reg_ development and its distribution to secondary lymphoid organs. We analyzed the activation status of T cells by staining CD44 and CD62L. This revealed that both CD4^+^ and CD8^+^ T cells from *Foxp3*^Δ*Adar1*^ mice exhibited a clear shift from a naïve (CD44^−^CD62L^+^) to an effector phenotype (CD44^+^CD62L^−^) (Fig. 2, I and J, and fig. S4, A and B). In contrast, the naïve and effector T cells in *Foxp3*^Δ*Trex1*^ mice were similar to those in littermate controls (fig. S4, C and D).

Defective control of T helper cell 2 (T_H_2)–related cytokines has been reported in the *scurfy* mutant (*39*); thus, we stimulated splenic CD4^+^ T cells using anti-CD3/anti-CD28 antibody–coated beads and monitored cytokine production. The *Foxp3*^Δ*Adar1*^ mutant cells showed higher production of T_H_2-related proinflammatory cytokines, such as IL-4, IL-5, IL-10, and IL-13 (Fig. 2K). Consistent with this, approximately 30% of CD4^+^ T cells from *Foxp3*^Δ*Adar1*^ mice were GATA3 positive compared to <1% of *Foxp3*-WT CD4^+^ T cells (Fig. 2L). These data indicate that *Foxp3*^Δ*Adar1*^ mice exhibit *scurfy* mouse–like lethal autoimmune symptoms as a consequence of T_reg_ loss and abnormal activation of effector T cells.

### Constitutive MDA5 activation in T_regs_ causes T_reg_ loss and autoimmunity in mice

**Fig. 3.**
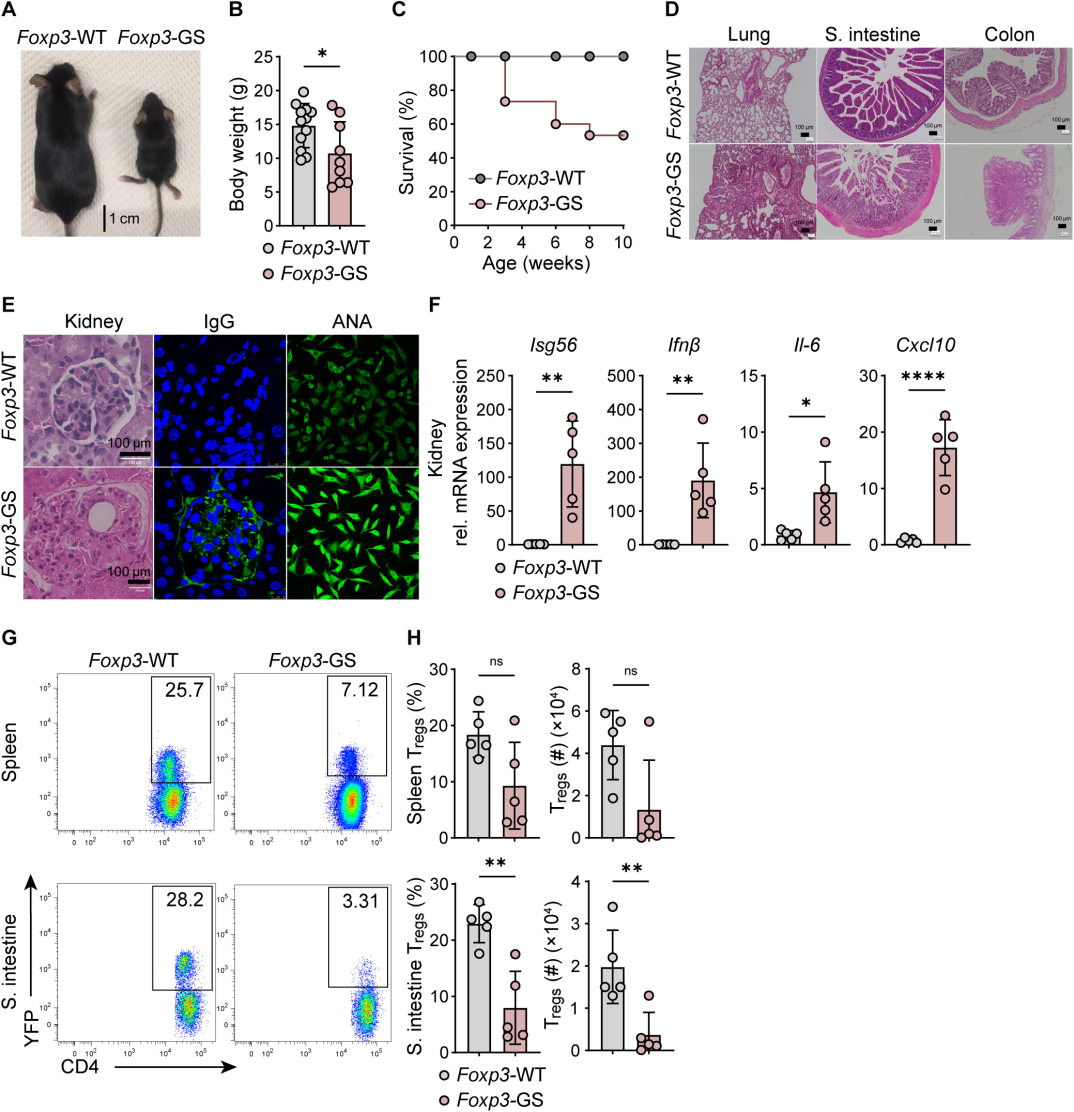
Constitutive MDA5 activation in T_regs_ causes T_reg_ loss and autoimmunity in mice. (**A** and **B**) Pictures and body weight measurements of 4-week-old *Foxp3*-WT (*n* = 12) and *Foxp3*-GS (*n* = 9) mice. (**C**) Survival graph of *Foxp3*-WT (*n* = 12) and *Foxp3*-GS (*n* = 14) mice. (**D**) Representative H&E staining images of indicated organs. Scale bars, 100 μm. (**E**) Representative H&E staining images of kidneys, immunofluorescence staining images of IgG (green) in the kidneys (4′,6-diamidino-2-phenylindole (DAPI), blue), and immunofluorescence staining of L929 cells using sera from *Foxp3*-WT and *Foxp3*-GS mice. (**F**) Relative mRNA expression of indicated genes. (**G** and **H**) Representative FC plots, summarized percentages (%), and total numbers (#) of CD4^+^YFP^+^(FOXP3^+^) T_regs_ in spleens and small intestines. Panels (F) to (H) are representative of ≥3 independent experiments with ≥3 mice per group. In dot plots, each symbol indicates an individual mouse. Statistics were calculated using Student’s *t* test; *P ≤ 0.05; **P ≤ 0.01; ****P ≤ 0.0001; ns, not significant, *P* > 0.05.

ADAR1 deficiency constitutively activates the cytoplasmic dsRNA sensor MDA5 (*10*). We hypothesized that the T_reg_-specific constitutive activation of MDA5, by expressing the gain-of function mutant G821S of the *Ifih1* gene (referred to as MDA5 G821S) (*40*), would result in T_reg_ population loss and the onset of autoimmune diseases, similar to that observed in *Foxp3*^Δ*Adar1*^ mice. To examine the effect of constitutive MDA5 signaling on T_regs_, we established a conditional MDA5 G821S expression system in mice (fig. S5A). In the absence of Cre recombinase, truncated, nonfunctional MDA5 proteins were expressed from the mutated allele by the insertion of a stop cassette, whereas MDA5 G821S was expressed by the deletion of the stop cassette in the presence of Cre recombinase (fig. S5B). As previously reported, mice that systemically express the MDA5 G821S mutant in this system exhibited severe growth retardation and autoimmune disorders including lupus-like nephritis (*40*). In the absence of Cre expression, MDA5 G821S^fl/+^ mice did not exhibit any phenotypes and were comparable to WT mice. Next, for the specific expression of MDA5 G821S in T_regs_, we intercrossed MDA5 G821S^fl/+^ mice with *Foxp3*^YFP-Cre^ mice to generate *Foxp3*^Cre/Cre^ MDA5 G821S^fl/+^ female or *Foxp3*^Cre/Y^ MDA5 G821S^fl/+^ male mice, herein referred to as *Foxp3*-GS mice. *Foxp3*-GS mice also exhibited growth retardation and reduced body weight compared to littermate *Foxp3*-WT mice (Fig. 3, A and B). Approximately 50% of the *Foxp3*-GS mice survived until 8 weeks after birth, and some survived until almost 1 year (Fig. 3C), indicating a milder disease progression than that observed in *Foxp3*^Δ*Adar1*^ mice, which did not survive >4 weeks after birth (Fig. 2C). We primarily used adult *Foxp3*-GS mice (aged 8 to 12 weeks) along with *Foxp3*-WT littermate controls for further analyses. Histologic evaluation of organs revealed that *Foxp3*-GS mice exhibited severe tissue inflammation with immune cell infiltration of the lungs, small intestine and colon, nephritis with immunoglobulin G (IgG) deposition, as well as the presence of antinuclear antibodies (ANAs) in the sera (Fig. 3, D and E). The up-regulation of *Isg56*, *Ifn*-β, *Il-6*, and *Cxcl10* was also detected in the kidneys (Fig. 3F). Notably, we did not detect ANAs in the sera of *Foxp3*^Δ*Adar1*^ mice, possibly due to their young age of 3 weeks old at the time of analysis (fig. S6). We subsequently examined the T_reg_ compartment in *Foxp3*-GS mice and found a reduction compared to littermate controls (Fig. 3, G and H). Although adult *Foxp3*-GS mice surviving >8 weeks generally exhibited milder loss of peripheral T_regs_ and autoimmune phenotypes than *Foxp3*^Δ*Adar1*^ mice, these data reveal that intrinsic and constitutive MDA5 signaling in T_regs_ leads to a reduction of the T_reg_ population and triggers the onset of autoimmune symptoms.

### *Adar1* deletion in T_regs_ activates the PKR/eIF-2α pathway contributing to cell death

**Fig. 4.**
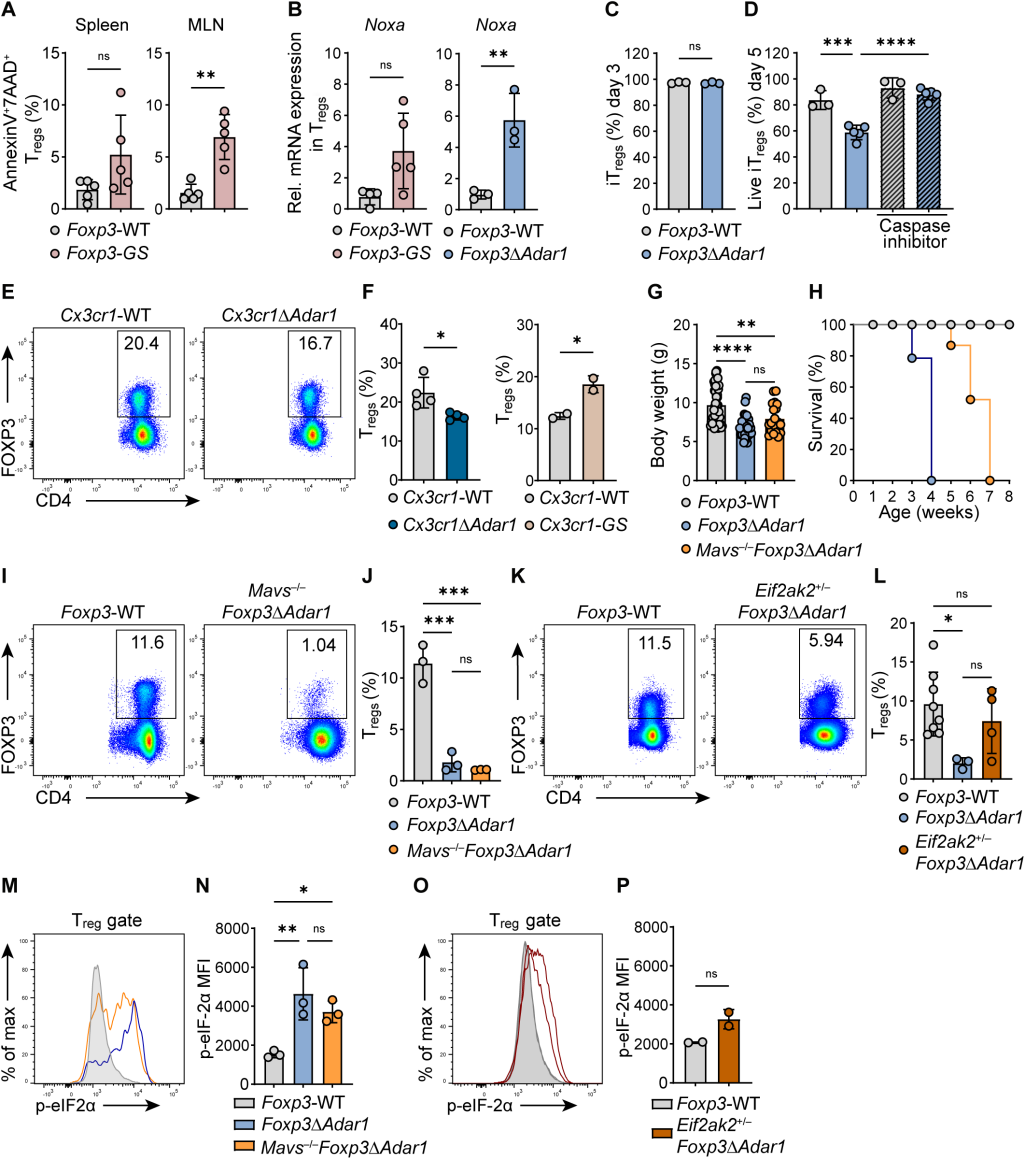
*Adar1* deletion in T_regs_ activates both the MDA5/MAVS and PKR/eIF-2α pathways contributing to cell death. (**A**) Annexin V^+^7AAD^+^ T_reg_ percentages in the spleens and mesenteric lymph nodes (MLNs). (**B**) Relative mRNA expression of *Noxa* in sorted CD4^+^YFP^+^(FOXP3^+^) T_regs_. (**C** and **D**) Percentages of CD4^+^FOXP3^+^ ex vivo induced T_regs_ (iT_regs_) at day 3 and their viability at day 5. iT_regs_ were induced from *Foxp3*-WT (*n* = 3) or *Foxp3*^ΔAdar1^ (*n* = 5) naïve CD4^+^ T cells and treated or not with caspase inhibitor. (**E** and **F**) Representative FC plots and summarized percentages of CD4^+^FOXP3^+^ T_regs_ in the spleens of 30-week-old *Cx3cr1*^Δ*Adar1*^, 10-week-old *Cx3cr1*-GS, and respective *Cx3cr1*-WT control mice. (**G**) Body weight measurements of 3-week-old *Foxp3*-WT (*n* = 44), *Foxp3*^Δ*Adar1*^ (*n* = 36), and *Mavs*^−/−^*Foxp3*^Δ*Adar1*^ (*n* = 20) mice. (**H**) Survival graphs of *Foxp3*-WT (gray), *Foxp3*^Δ*Adar1*^ (blue, *n* = 7), and *Mavs*^−/−^*Foxp3*^Δ*Adar1*^ mice (orange, *n* = 5). (All *Foxp3*^Δ*Adar1*^ and *Mavs*^−/−^*Foxp3*^Δ*Adar1*^ mice to date developed a severe phenotype and were sacrificed at latest 4 or 8 weeks old, respectively.) (**I** to **L**) Representative FC plots and summarized percentages of CD4^+^FOXP3^+^ T_regs_ in the spleens. (**M** to **P**) Representative histograms and plots of phospho–eIF-2α mean fluorescence intensity in CD4^+^FOXP3^+^ T_regs_ from spleens. Panels (A), (B), (I), (J), (M), and (N) are representative of ≥3 independent experiments with ≥3 mice per group; panels (C) to (F) are representative of two independent experiments with ≥2 mice per group. In dot plots, each symbol indicates an individual mouse. Statistics were calculated using Student’s *t* test or one-way ANOVA; *P ≤ 0.05; **P ≤ 0.01; ***P ≤ 0.001; ****P ≤ 0.0001; ns, not significant, *P* > 0.05.

Next, we investigated whether cell death is involved in T_reg_ reduction in *Foxp3*-GS and *Foxp3*^Δ*Adar1*^ mice. Flow cytometric analysis of annexin V and 7-aminoactinomycin D (7AAD) revealed that *Foxp3*-GS mice exhibited a higher frequency of apoptotic T_regs_ than control *Foxp3*-WT mice (Fig. 4A). We also found up-regulated mRNA expression of the pro-apoptotic gene *Noxa* in CD4^+^YFP^+^(FOXP3^+^) T_regs_ sorted from the spleens of *Foxp3*-GS and *Foxp3*^Δ*Adar1*^ mice compared to controls (Fig. 4B). The expression of another pro-apoptotic gene *Puma*, as well as *Isg56*, was also up-regulated in *Foxp3*-GS T_regs_, while the expression of other pro-apoptotic BH3-only genes, including *Bim* and *Bad*, and anti-apoptotic genes *Bcl-2*, *Mcl-1*, and *Bcl-xL* was comparable (fig. S7). Because the T_reg_ population in *Foxp3*^Δ*Adar1*^ mice is extremely reduced, we induced T_reg_ differentiation by culturing enriched naïve CD4^+^ T cells with IL-2 and transforming growth factor–β (TGF-β) to further examine the apoptotic events. Ex vivo induced T_regs_ (iT_regs_) from *Foxp3*^Δ*Adar1*^ mice expressed FOXP3 similar to *Foxp3*-WT iT_regs_ but showed significant cell death that was rescued by treatment with a pan-caspase inhibitor, Q-VD-OPH (Fig. 4, C and D). To further investigate the effect of extrinsic factors on T_regs_, we examined mice with *Adar1* deletion or MDA5 G821S mutant expression specifically in CX3CR1-positive immune cells, indicated as *Cx3cr1*^Δ*Adar1*^ and *Cx3cr1*-GS. We found that the FOXP3^+^ T_reg_ population was only mildly affected in both models. Notably, we confirmed ISG signature in the spleen of *Cx3cr1*-GS mice (Fig. 4, E and F, and fig. S8). These data indicate that intrinsic signaling in T_regs_ caused by ADAR1 deficiency or chronic MDA5 activation leads to apoptotic cell death and loss of the T_reg_ population, in both *Foxp3*-GS and *Foxp3*^Δ*Adar1*^ mice.

Given that concurrent deletion of *Ifih1* and/or *Mavs* delays the embryonic lethality of systemic *Adar1*^−/−^ mice (*10*–*12*), we examined a potentially similar effect and generated *Mavs*^−/−^*Foxp3*^Δ*Adar1*^ mice. At the age of 3 weeks, *Mavs*^−/−^*Foxp3*^Δ*Adar1*^ mice showed improved body weight and appearance(Fig. 4G and fig. S9A), and significant reduction of inflammatory cytokines in their kidneys, compared to age-matched *Foxp3*^Δ*Adar1*^ mice (fig. S9B). However, their condition deteriorated at approximately 4 weeks after birth, and they died by 8 weeks after birth (Fig. 4H). Although *Mavs*^−/−^*Foxp3*^Δ*Adar1*^ mice exhibited mild improvement in terms of growth retardation and survival compared to *Foxp3*^Δ*Adar1*^ mice, at 3 weeks old, they already showed a significant loss of T_regs_ (Fig. 4, I and J, and fig. S9, C to E) and a significant shift from naïve to effector CD4^+^ and CD8^+^ T cells, similar to that observed in *Foxp3*^Δ*Adar1*^ mice (fig. S9F), indicating that an MDA5-independent pathway is also critically involved in T_reg_ homeostasis in *Foxp3*^Δ*Adar1*^ mice.

It is known that ADAR1 deficiency activates PKR encoded by *Eif2ak2* (*13*), and to examine its involvement in the T_reg_ cell death, we intercrossed *Foxp3*^Δ*Adar1*^ mice with *Eif2ak2*^−/−^ knockout mice. *Foxp3*^Δ*Adar1*^ mutant mice with systemic heterozygous PKR deficiency, *Eif2ak2*^+/−^*Foxp3*^Δ*Adar1*^, showed an improvement of their appearance and body weight; especially the skin of their ears, tails, and overall fur coat were comparable to those of littermate controls, in contrast to the severe *scurfy*-like appearance of age-matched *Foxp3*^Δ*Adar1*^ mutant mice (fig. S10, A and B). *Eif2ak2*^+/−^*Foxp3*^Δ*Adar1*^ mice retained a larger fraction of T_regs_, compared to that observed in *Foxp3*^Δ*Adar1*^ mutant mice (Fig. 4, K and L). We also observed a down-regulation of proinflammatory cytokines and ISGs in organs such as kidney and liver (fig. S10C). PKR activation leads to eIF-2α phosphorylation, which subsequently blocks protein synthesis (*13*). The T_reg_ population in *Foxp3*^Δ*Adar1*^ and *Mavs*^−/−^*Foxp3*^Δ*Adar1*^ mice exhibited high phosphorylation status of eIF-2α compared to controls (Fig. 4, M and N). The intensity of phosphorylated eIF-2α in T_regs_ from *Eif2ak2*^+/−^*Foxp3*^Δ*Adar1*^ mice was comparable to controls and significantly reduced compared to T_regs_ from *Foxp3*^Δ*Adar1*^ mice (Fig. 4, O and P, and fig. S10D). Moreover, protein synthesis capacity monitored by puromycin incorporation was lower in T_regs_ from *Foxp3*^Δ*Adar1*^ mice compared to controls (fig. S11, A and B). These data show evidence of PKR/eIF-2α–dependent protein synthesis shutoff, independently of MAVS signaling. ADAR1 deficiency also activates RNase L, leading to cell death (*14*); however, we did not observe 28*S* ribosomal RNA (rRNA) cleavage in RNA isolated from T_regs_ of *Foxp3*^Δ*Adar1*^ mice, indicating that the OAS/RNase L pathway was not activated in these cells (fig. S12). Our data suggest that both MDA5/MAVS and PKR/eIF-2α pathways are involved in the dysregulation of T_reg_ homeostasis in *Foxp3*^Δ*Adar1*^ mice and that ablation of the MDA5/MAVS pathway is not sufficient to rescue their phenotype.

## DISCUSSION

We found a reduction of the T_reg_ population in patients with AGS caused by *ADAR1* or *IFIH1* mutations. In particular, the suppressive T_reg_ population in Fr. II was significantly reduced, and this T_reg_ population also significantly up-regulated the expression of PD-1, which may cause attenuation of T_reg_ function (*35*, *37*). Together with murine data, we demonstrate the concept that dysregulated innate immune signaling due to ADAR1 deficiency or chronic MDA5 activation in T_regs_ is sufficient to cause autoimmunity as a consequence of T_reg_ loss. Thus, our findings indicate that along with constitutive IFN-I activation, T_reg_ loss and/or attenuation of T_reg_ function (*25*) are critically involved in the onset of autoimmune disease, and that systemic or local T_reg_ dysregulation may explain why patients with type I interferonopathies exhibit a variety of autoimmune manifestations. Considering that there are approximately 40 distinct genes associated with type I interferonopathies (*1*), it would be of interest to examine the T_reg_ populations in patients carrying mutations other than *ADAR1* or *IFIH1*.

The limited availability of samples from patients with AGS or other type I interferonopathies, as well as the generally low frequency of T_regs_ in PBMCs, prompted us to use two murine models for further characterization. We found that ADAR1 deficiency leading to chronic activation of innate immune sensors MDA5 and PKR, as well as expression of constitutively active gain-of-function MDA5 in T_regs_, induce apoptotic cell death and loss of peripheral T_regs_, resulting in highly lethal autoimmune phenotypes. While we previously observed reduced T_reg_ total numbers in mice that systemically express the MDA5 G821S mutant protein (*25*), the T_reg_ numbers were only mildly affected in both *Cx3cr1*^Δ*Adar1*^ and *Cx3cr1*-GS mice with ISG signature, indicating that the T_reg_ cell death is caused by intrinsic signaling rather than extrinsic effects from other immune cells. Several models with T_reg_-specific deletion of anti-apoptotic genes such as MCL-1 and c-FLIP have been reported (*41*, *42*), which cause similar T_reg_ loss and lead to lethal autoimmune phenotypes. Moreover, we demonstrate that ADAR1 deficiency in T_regs_ triggers PKR/eIF-2α–dependent protein synthesis shutoff, which is likely the major driving force toward cell death, given the severer and earlier onset of phenotype in *Foxp3*^Δ*Adar1*^ mice compared to that of *Foxp3*-GS mice, as well as the larger frequency of T_regs_ retained in *Eif2ak2*^+/−^*Foxp3*^Δ*Adar1*^ mice. However, further investigation is needed to determine the long-term outcome of, especially homozygous, *Eif2ak2* deletion in *Foxp3*^Δ*Adar1*^ mice. It has been recently shown that simultaneous deletion of *Ifih1* and *Eif2ak2* is necessary to rescue the embryonic lethality of systemic ADAR1 p150-isoform knockout mice (*16*), suggesting that ablation of both RNA sensing pathways could be required to likewise rescue the *Foxp3*^Δ*Adar1*^ mutant mice.

ADAR1 deficiency has also been reported to trigger ZBP1-induced necroptosis (*15*, *43*–*46*) and p16-dependent senescence (*47*); therefore, the potential involvement of these mechanisms in the T_reg_ loss in this T_reg_-specific ADAR1 deficient mouse model should be explored. Recently, mice with AGS-related *Adar1* mutations have been reported to exhibit MDA5-dependent severe inflammation and AGS-like encephalopathy (*48*, *49*). It is of interest to explore whether T_regs_ are affected by the respective mutations and their potential involvement in disease in these mice.

JAK inhibitors are used to treat type I interferonopathies, specifically aiming to reduce chronic IFN-I signaling, and they are beneficial to some extent, for example, in reducing inflammation and ameliorating skin lesions (*50*). JAK inhibitors reported to treat type I interferonopathies, so far, are not selective for individual JAKs. Moreover, considering that JAKs are critical in the signaling of different immunologically essential pathways, JAK inhibition has a broad immunosuppressive effect, leading to the increased risk of viral infections (*51*). JAK inhibitors also down-regulate FOXP3 expression in vitro and in vivo (*31*), and some studies have revealed that JAK inhibition resulted in a stark and long-lasting reduction of peripheral T_regs_ (*52*). Reducing the IFN-I response is currently the primary target for treating patients with type I interferonopathies, and there is a need for more precise inhibitors. However, it should also be considered that IFN-I is not the only culprit driving the pathogenesis and that its inhibition is insufficient to alleviate already developed autoimmune symptoms. T_reg_ adoptive transfer therapies have shown promising outcomes, for instance, in patients with type 1 diabetes (*53*) or in patients with amyotrophic lateral sclerosis, which is a neurological disease wherein FOXP3-expressing cells decrease with disease progression (*54*). On the basis of our findings, it is worthwhile to perform an in-depth functional analysis of T_regs_ in patients with type I interferonopathies caused by aberrant innate immune sensing, to potentially use them in combination with available treatments to improve clinical manifestations.

## MATERIALS AND METHODS

### Ethical statement

Blood samples were obtained with informed consent from patients with AGS and healthy donors, with approval from the Medical Ethics Committee of Kyoto University School of Medicine (R2831-2) and from the Ethics Committee of University of Dresden (TRR237/A11).

### Collection and analysis of PBMCs

PBMCs were isolated from the buffy coats or whole blood samples via Ficoll-Paque density gradient centrifugation (GE Healthcare, #17-1440-02).

### Mice

All animal experiments were performed according to the institutional and governmental guidelines of animal welfare in Germany (81-02.04.2019.A462) and Japan (Kyoto, 19323). *Adar1*^flox^ mice (*55*) (B6.129-*Adar^tm1Knk^*/Mmjax, The Jackson Laboratory, #034619-JAX) were intercrossed with *Foxp3*^YFP-Cre^ mice (*56*) (B6.129(Cg)-*Foxp3^tm4(YFP/icre)Ayr^*/J, The Jackson Laboratory, #016959) to generate homozygous male *Foxp3*^Cre/Y^ *Adar1*^fl/fl^ or female *Foxp3*^Cre/Cre^ *Adar1*^fl/fl^ mice with *Adar1* deletion in FOXP3-expressing T cells, indicated as *Foxp3*^ΔAdar1^ or *Foxp3*Δ*Adar1*. *Trex1*^flox^ mice were generated as previously described (*57*) and were intercrossed with *Foxp3*^YFP-Cre^ mice to generate homozygous male *Foxp3*^Cre/Y^ *Trex1*^fl/fl^ or female *Foxp3*^Cre/Cre^ *Trex1*^fl/fl^ mice with *Trex1* deletion in FOXP3-expressing T cells, indicated as *Foxp3*^Δ*Trex1*^ or *Foxp3*Δ*Trex1*. *Mavs*^−/−^ mice (*58*) were provided by S. Akira (Osaka University, Suita, Japan). These mice were intercrossed with *Foxp3*^Δ*Adar1*^ mice to generate male *Mavs*^−/−^*Foxp3*^Cre/Y^ *Adar1*^fl/fl^ or female *Mavs*^−/−^*Foxp3*^Cre/Cre^ *Adar1*^fl/fl^ mice, indicated as *Mavs*^−/−^*Foxp3*^Δ*Adar1*^ or *Mavs*^−/−^*Foxp3*Δ*Adar1*. *Pkr*^−/−^ mice (*59*) (here indicated as *Eif2ak2^−/−^* mice) were provided by F. Weber (University of Giessen, Germany) with agreement from J. Pavlovic (University of Zurich, Switzerland) and were intercrossed with *Foxp3*^Δ*Adar1*^ to generate *Eif2ak2*^+/−^*Foxp3*^Δ*Adar1*^ mice.

To generate conditional MDA5 G821S^fl/+^ mice, a target construct containing loxP, an exon 13 fragment, a stop codon, poly A, and a PGK-Neo cassette with loxP sites was used; it was linked with the mutant exon 13 G821S (fig. S5A). The sequences containing a missense mutation in MDA5 exon 13 were amplified by polymerase chain reaction (PCR) and then inserted into the above construct. Then, the linearized targeting vector was transduced into murine hybrid embryonic stem (ES) cells via electroporation. Northern blotting was performed to confirm successful recombination in ES cells. Chimeric mice were bred with C57BL/6J mice for germline transmission (MDA5 G821S^fl/+^ mice). MDA5 G821S^fl/+^ mice were crossed with *Foxp3*^YFP-Cre^ mice to generate male *Foxp3*^Cre/Y^ MDA5 G821S^fl/+^ or female *Foxp3*^Cre/Cre^ MDA5 G821S^fl/+^ mice, indicated as *Foxp3*-GS mice. *Adar1*^flox^ mice were intercrossed with *Cx3cr1*^Cre^ mice (*60*) [B6J.B6N(Cg)-*Cx3cr1^tm1.1(cre)Jung^*/J, The Jackson Laboratory, #025524] to generate *Cx3cr1*^Cre^ *Adar1*^fl/fl^ mice with *Adar1* deletion specifically in CX3CR1-expressing immune cells, indicated as *Cx3cr1*^Δ*Adar1*^ or *Cx3cr1*Δ*Adar1*. *Cx3cr1*^Cre^ mice were intercrossed with MDA5 G821S^fl/+^ mice to generate *Cx3cr1*^Cre^ MDA5 G821S^fl/+^ mice, indicated as *Cx3cr1*-GS.

### Single-cell suspensions

Mouse spleens or lymph nodes were passed through 100-μm cell strainers (Sigma-Aldrich, #CLS431752-50EA) in fluorescence-activated cell sorting (FACS) buffer [phosphate-buffered saline (PBS; Thermo Fisher Scientific, #10010056) containing 5% fetal bovine serum (FBS) (Thermo Fisher Scientific, #10270106) and 2 mM EDTA (Merck, #93283)], incubated with ammonium-chloride-potassium lysing buffer (Thermo Fisher Scientific, #A1049201) for up to 5 min to lyse red blood cells, and then filtered once again through 70-μm cell strainers. To obtain single-cell suspensions from the small intestine, Peyer’s patches were first removed, and then the intestine was washed thoroughly with cold PBS and incubated in RPMI 1640 containing 3% FBS, 100 mM dithiothreitol (Thermo Fisher Scientific, #20290), and 0.5 mM EDTA, with shaking for 20 min at 37°C. After washing several times, the intestines were cut into small pieces and digested with deoxyribonuclease (0.5 mg/ml; Roche, #04716728001) and Liberase TL (1 mM/ml; Roche, #05401020001) in RPMI 1640 for 23 min at 37°C. The obtained single cells were filtered through 70-μm nylon screens in FACS buffer.

### Cell culture and stimulation

CD4^+^ or naïve CD4^+^ T cells were isolated from whole splenocyte suspensions via negative selection (Miltenyi Biotec, #130-104-454 and #130-104-453) and cultured in RPMI medium (Thermo Fisher Scientific, #21875091) containing 10% FBS (Thermo Fisher Scientific, #10270106), 1× minimum essential medium nonessential amino acids (Gibco, #11140-035), 2 mM l-glutamine (Gibco, #25030-024), 1 mM sodium pyruvate (Gibco, #11360-039), penicillin (100 U/ml)–streptomycin (100 μg/ml) (Gibco, #15140-122), 25 mM Hepes (Pan-Biotech, #P05-01100), and 0.05 mM β-mercaptoethanol (Pan-Biotech, #P07-05020).

To induce T_reg_ differentiation, naïve CD4^+^ T cells were cultured on plates coated with α-CD3 (1 μg/ml) and α-CD28 (5 μg/ml) (eBioscience, #16-0031-81 and #16-0281-81) in the presence of IL-2 (50 ng/ml; BioLegend, #575406) and TGF-β (10 ng/ml; PeproTech, #100-21). The cells were then treated with 1 μM Q-VD-OPH pan-caspase inhibitor (MedChemExpress, #HY-12305). For enzyme-linked immunosorbent assay (ELISA; BioLegend, #740741), the enriched CD4^+^ T cells were stimulated overnight with mouse T activator CD3/CD28 beads (Thermo Fisher Scientific, #11-453-D) in a bead-to-cell ratio of 1:2. ELISA was performed using the LEGENDplex Mouse Th Cytokine Panel (BioLegend, #740741) according to the manufacturer’s instructions. To assess protein synthesis capacity, CD4^+^ T cells enriched by negative selection (Miltenyi Biotec, #130-104-454) were incubated with puromycin (10 μg/ml; Sigma-Aldrich, #P7255) for 45 min at 37°C, 5% CO_2_ atmosphere, then stained intracellularly using the eBioscience Foxp3/Transcription Factor Staining Buffer Set (Invitrogen, #00-5523), and analyzed by flow cytometry.

### Flow cytometry analysis

To assess cell viability, the cells were stained with fixable live/dead staining dye (BioLegend, #423114) diluted in PBS or 7AAD viability staining solution (BioLegend, #420403) for 15 min at room temperature (RT) protected from light. Thereafter, the cells were washed with PBS and incubated with Fc block diluted in FACS buffer (InVivoMAb anti-mouse CD16/CD32, Bio X Cell, #BE0307) for 15 min at 4°C protected from light. For surface staining, the cells were incubated with the desired antibody mix in FACS buffer for 20 to 30 min at 4°C protected from light. For intracellular staining, the cells were fixed and permeabilized using either the eBioscience Foxp3/Transcription Factor Staining Buffer Set (Invitrogen, #00-5523) for transcription factor staining or the BD Cytofix/Cytoperm Kit (#554714) for cytosolic staining. Then, the cells were incubated with the desired antibody mix in corresponding 1× wash buffer for 20 to 30 min at 4°C protected from light. Anti–phospho–eIF-2α staining was performed using the eBioscience Foxp3/Transcription Factor Staining Buffer Set (Invitrogen, #00-5523), followed by staining with a secondary antibody conjugated to the preferred fluorochrome diluted in 1× wash buffer. The cells were then analyzed or sorted either on the BD Canto II, BD LSRFortessa, BD FACSAria Fusion, or BD FACSAria III systems and further analyzed using FlowJo Software (BD Biosciences). The anti-mouse antibodies used were as follows: CD4-allophycocyanin (APC) (RM4-5, BioLegend, #100516), CD44-peridinin chlorophyll protein (PerCP)/cyanine 5.5 (Cy5.5) (IM7, BioLegend, #103032), CD62L-phycoerythrin (PE) (MEL-14, BioLegend, #10407), Foxp3–Alexa Fluor 488 (AF488) (150D, BioLegend, #320012), green fluorescent protein (GFP)–AF488 (FM-264G, BioLegend, #338008), CD8–Brilliant Violet 650 (BV650) (53-6.7, BioLegend, #100741), Gata3-PE (16E10A23, BioLegend, #653803), GFP-AF488 (FM264G, BioLegend, #338008), phospho–eIF-2α (Ser^51^, Cell Signaling Technology, #3597), active caspase-3–PE (C92-605, BD, #561011), annexin V–PE-Cy7 (BioLegend, #640950), anti-rabbit IgG-PE (Cell Signaling Technology, #79408), and puromycin AF647 (Sigma-Aldrich, #MABE343-AF647). The anti-human antibodies used were as follows: CD4-APC (SK3, BioLegend, #344614), CD45RA-PerCP/Cy5.5 (HI100, BioLegend, #304122), CD25–PE-Cy7 (BC96, BioLegend, #302612), CD25-PE (M-A251, BD, #560989), CD152 (CTLA-4)–PE-Cy7 (BNI3, BioLegend, #369614), and CD279 (PD-1)-PE/Dazzle594 (EH12.2H7, BioLegend, #329940).

### Histological staining

The organs were fixed with 4% paraformaldehyde (PFA) in PBS solution (Thermo Scientific Chemicals, J19943.K2) and then embedded with paraffin. Thereafter, 3-μm sections were prepared and stained with hematoxylin and eosin (H&E) using standard protocols.

### IgG staining

First, 3-μm kidney sections were incubated with proteinase K (Invitrogen, #25530049) for antigen retrieval. Then, the sections were washed thrice (for 5 min each time) with PBS and incubated with PBS containing 20% donkey serum (Sigma-Aldrich, #D9663) and 0.05% Triton X-100 (Carl Roth, #3051.4) for 1 hour at RT. Thereafter, the sections were stained with IgG (Jackson ImmunoResearch, #715-606-151) diluted 1:50 in PBS containing 10% donkey serum for 1 hour at RT protected from light. After washing the sections once (1 min) with PBS, the sections were incubated with 4′,6-diamidino-2-phenylindole (Invitrogen, #D1306) diluted 1:1000 in PBS for 5 min at RT protected from light, followed by washing thrice (1 min each time) with PBS. The samples were imaged on the SP8 LIGHTNING confocal microscope (Leica).

### ANA detection

L929 cells were seeded into an eight-well chamber (ibidi, #80826) and cultured overnight at 37°C in a 5% CO_2_ atmosphere. Thereafter, the cells were washed with PBS and fixed with 4% PFA in PBS solution for 10 min at RT, washed again with PBS, and permeabilized with 0.1% Triton X-100 in PBS (PBST) for 20 min at RT. Then, the cells were incubated with blocking buffer [0.5% normal goat serum (Abcam, #ab7481) in PBST] for 1 hour at RT and further incubated with previously isolated mouse serum (diluted 1:150 in PBST) overnight at 4°C. The cells were washed twice with PBST and incubated with anti-mouse IgG (goat) secondary antibody diluted 1:1000 in PBST. After washing twice with PBST, the cells were kept in PBS and imaged on the SP8 LIGHTNING confocal microscope (Leica).

### Assessment of RNA degradation

To assess RNA status, RNA was extracted from sorted CD4^+^YFP^+^ T cells using the Direct-Zol RNA Microprep Kit (Biozym, #R2061) and then analyzed using the Agilent 2200 TapeStation using High-Sensitivity RNA ScreenTape and Reagents according to the manufacturer’s instructions (Agilent Technologies, #5067-5579, #5067-5580, and #5067-5581).

### Quantitative reverse transcription PCR

Whole organs were collected in TRIzol (Invitrogen, #15596) and homogenized using the gentleMACS tissue dissociator and tubes (Miltenyi Biotec, #130-096-427 and #130-093-237). Then, RNA was extracted using the phenol-chloroform method (Panreac AppliChem, #A1153,0100 and #A3691,1000). cDNA was generated using the High-Capacity cDNA Reverse Transcription Kit (Applied Biosystems, #43688). Reverse transcription PCR was performed using the Fast SYBR Green Master Mix or TaqMan Fast Advanced Master Mix on the Step One Plus Real-Time PCR System (Applied Biosystems, #4385614, #4444558, and #4376600). Relative RNA expression was determined using the ΔΔ*C*_T_ method, in which ΔΔ*C*_T_ = Δ*C*_T_ of the target gene from the sample of interest (e.g., *Cxcl10* from *Foxp3*^Δ*Adar1*^) − Δ*C*_T_ of the same gene from the control sample (e.g., *Cxcl10* from *Foxp3*-WT), and Δ*C*_T_ = *C*_T_ (of the target gene) − 18*S* rRNA *C*_T_ (from the respective sample). The TaqMan probes (Thermo Fisher Scientific, #4331182) used were as follows: *Isg56* (*Ifit1*) (#Mm00515153_m1), *Ifn*β (#Mm00439546_s1), *Il-6* (#Mm01210733_m1), and *Cxcl10* (#Mm00445235_m1). The SYBR oligonucleotides used were as follows: *Noxa* [forward (F)–5′-GGAGTGCACCGGACATAACT-3′ and reverse (R)–5′-TTGAGCACTCGTCCTTCA-3′], *Puma* (F-5′-TGCTCTTCTTGTCTCCGCCG-3′ and R-5′-CATAGAGCCACATGCGAGCG-3′), *Bad* (F-5′-CGAAGGAGCGATGAGTT-3′ and R-5′-CCCACCAGGACTGGATAATG-3′), *Bim* (F-5′-GCCAAGCAACCTTCTGATGT-3′ and R-5′-CTGTCTTGCGGTTCTGTCTG-3′), *Bcl-2* (F-5′-GGTCTTCAGAGAGACAGCCAGGAGAAATC-3′ and R-5′-GTGGTGGAGGAACTCTTCAGGATG-3′), *Mcl-1* (F-5′-AAGCCAGCAGCACATTTCTGATGCC-3′ and R-5′-GTAATGGTCCATGTTTTCAAAGATG-3′), and *Bcl-xL* (F-5′-ACCAGCCACAGTCATGCCCGTCAGG-3′ and R-5′-GTAGTGAATGAACTCTTTCGGGAATGG-3′).

### Statistical analysis

Statistical analysis was performed using GraphPad Prism version 9 (9.5.1) for Windows (GraphPad Software, San Diego, CA, USA; www.graphpad.com). Student’s *t* test was used to compare the means of two groups, and ordinary one-way analysis of variance (ANOVA) was used to compare the means of three groups. *P* values are indicated as follows: *P ≤ 0.05; **P ≤ 0.01; ***P ≤ 0.001; ****P ≤ 0.0001; ns, not significant, *P* > 0.05.
