# Supplementary material for: Aberrant RNA sensing in regulatory T cells causes systemic autoimmunity
Source: Sci Adv. 2024 Mar 1;10(9):eadk0820. doi: 10.1126/sciadv.adk0820 (PMC10906915; doi:10.1126/sciadv.adk0820)
Supplement: Supplementary file 1 — Figs. S1 to S12 Table S1 [file sciadv.adk0820_sm.pdf]

Science Advances
AAAS

Supplementary Materials for

Aberrant RNA sensing in regulatory T cells causes systemic autoimmunity

Domnica Luca *et al.*

Corresponding author: Hiroki Kato, hkato@uni-bonn.de

Sci. Adv. **10**, eadk0820 (2024)
DOI: 10.1126/sciadv.adk0820

This PDF file includes:

Figs. S1 to S12
Table S1

Supplementary Materials

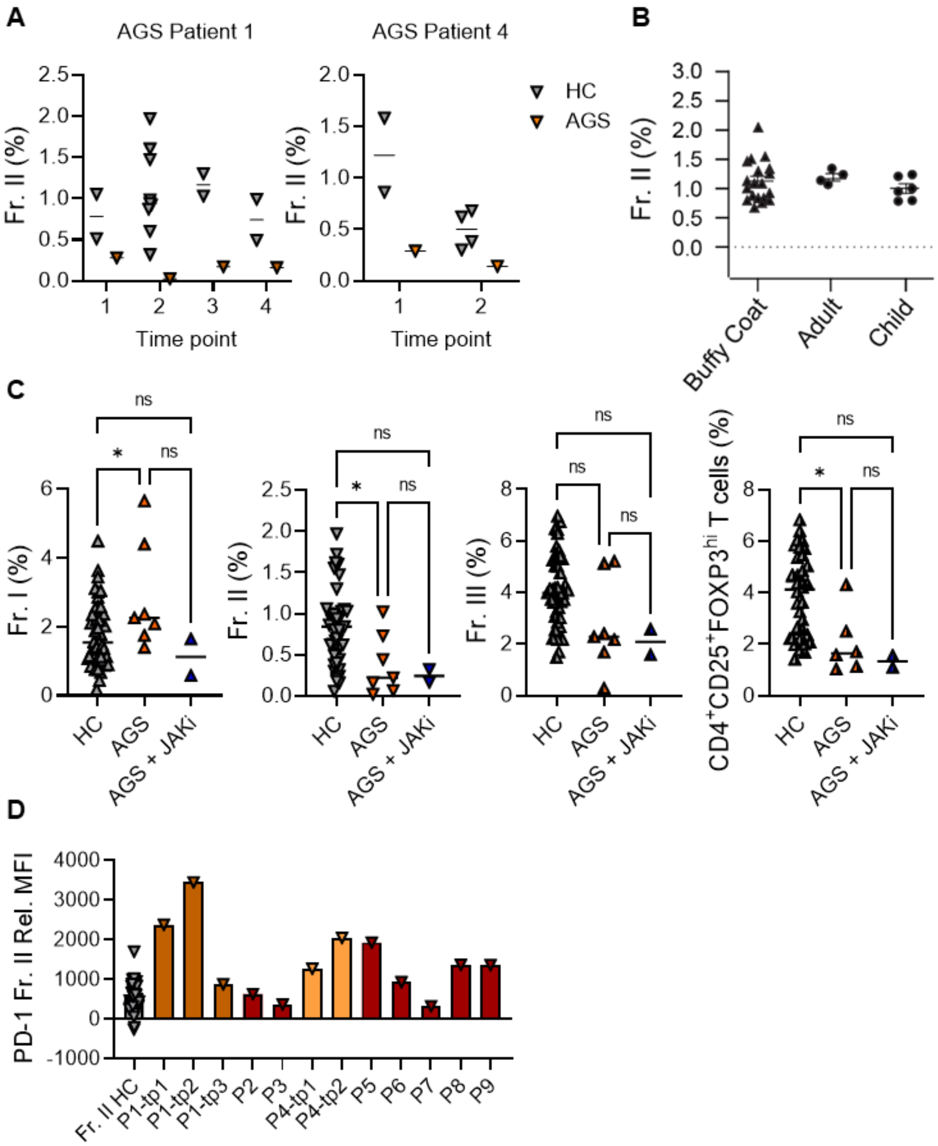

Fig. S1. Analysis of Treg populations in untreated and JAK inhibitor-treated patients with AGS. (**A**) Fr. II effector Treg percentages (%) ($CD25^{hi}CD45RA^{-}$ highly suppressive effector Tregs) in PBMCs from patients 1 and 4 with AGS (AGS), at time points 1–4 and 1 and 2, respectively, compared with corresponding healthy control samples (HC). (**B**) Fr. II of effector Treg percentages in PBMCs in buffy coats and whole blood samples from healthy adults (20–50 years old) and children (2–18 years old). (**C**) Percentages of Fr. I–III and $CD4^{+}CD25^{+}Foxp3^{hi}$ in PBMCs isolated from healthy control samples, untreated (AGS) and JAK inhibitor-treated patients with AGS (AGS + JAKi). (**D**) MFI values of PD-1 on Fr. II of effector Tregs, from healthy donors and each patient analysed (including 3 time points for patient 1 and 2 time points for patient 4), relative to MFI values of Fr. I from healthy controls. The samples from patients with AGS have been analysed one at a time (in two instances – two at a time), together with control samples from healthy donors. The dot plots shown here contain pooled data from respective analyses. Samples from patients 1 and 4 have been analysed at 4 (3 for CTLA-4 and

PD-1 expression – Fig. 1D and 1F, S1D) and 2 different time points (A and B), and the mean is represented as one symbol in pooled-data dot plots (C); otherwise, each symbol represents one individual. 3 and 2 different time points in (E) are represented as mean in (Fig. 1F). Statistics were calculated using one-way ANOVA; *, $p \leq 0.05$; ns, not significant, $p > 0.05$.

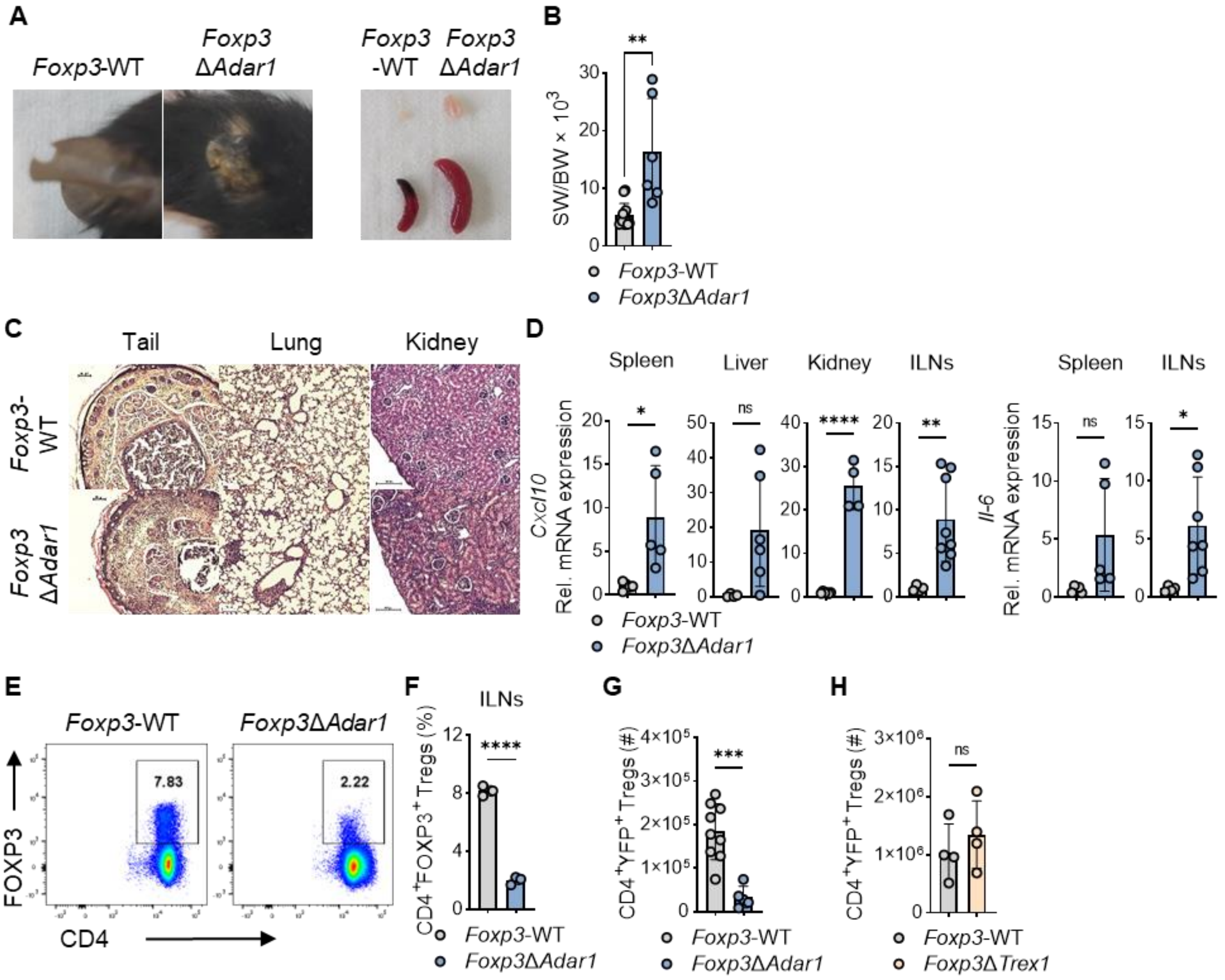

Fig. S2. Organ inflammation and Treg loss in $Foxp3^{\Delta Adar1}$ mice. (**A**) Representative pictures showing thickening of the skin (ears) and enlarged spleen and inguinal lymph node (ILN) in $Foxp3^{\Delta Adar1}$ mice. (**B**) Spleen weight (SW) by body weight (BW) ratio of $Foxp3^{\Delta Adar1}$ mice compared to controls. (**C**) Representative hematoxylin and eosin (H&E) staining images (scale bar=100µm) and (**D**) relative mRNA expression of *Cxcl10* and *Il-6* in indicated organs. (**E** and **F**) Representative flow cytometry plots and summarized percentages of CD4$^+$FOXP3$^+$ Tregs in inguinal lymph nodes (ILNs). (**G** and **H**) Total numbers of CD4$^+$FOXP3$^+$(YFP$^+$) Tregs in the spleens. Data are representative of ≥3 independent experiments with ≥3 mice per group. In dot plots, each symbol represents an individual mouse. Statistics were calculated using Student's t-test; *, $p \leq 0.05$; **, $p \leq 0.01$; ***, $p \leq 0.001$; ****, $p \leq 0.0001$; ns, not significant, $p > 0.05$.

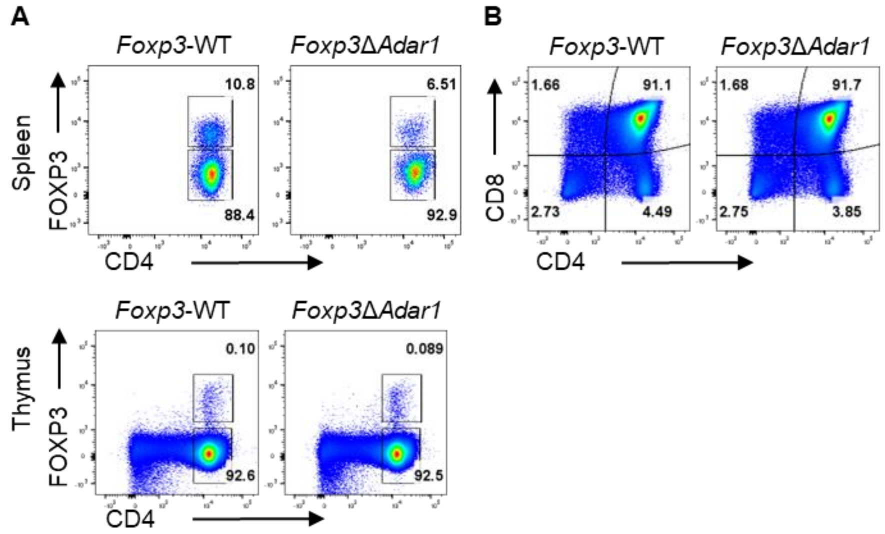

Fig. S3. Splenic and thymic Treg and CD4/CD8 T cell populations in 1-week-old *Foxp3*$^{\Delta Adar1}$ mice. (**A**) Representative flow cytometry analysis of CD4^{+}FOXP3^{+} Tregs in the spleen and thymus, and (**B**) CD4^{+}/CD8^{+} T cells in the thymus of 1-week-old *Foxp3*$^{\Delta Adar1}$ mice. Data are representative of 2 independent experiments with 2 *Foxp3*$^{\Delta Adar1}$ mice and >2 *Foxp3*-WT control mice.

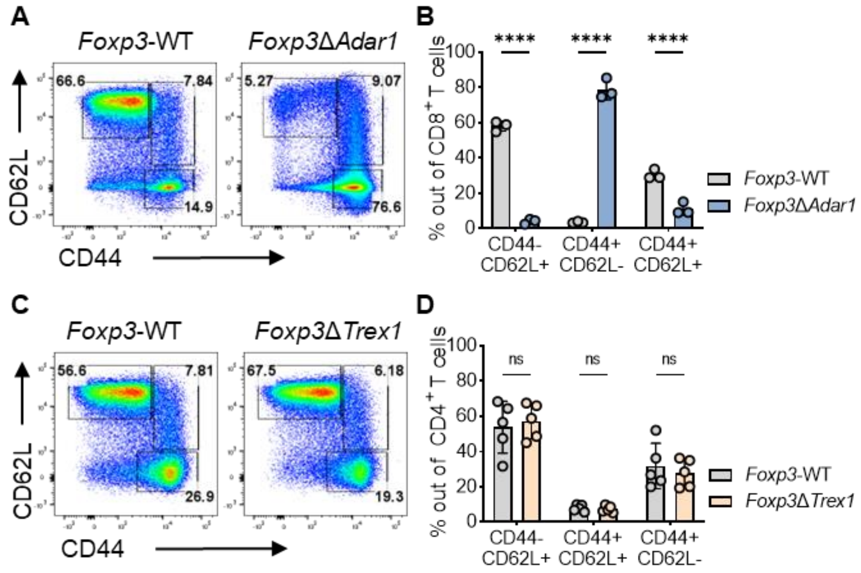

Fig. S4. Increased percentages of effector T cells in *Foxp3*$^{\Delta Adar1}$ but not in *Foxp3*$^{\Delta Trex1}$ mice. Representative flow cytometry plots and summarized percentages of naïve (CD44^{-}CD62L^{+}), effector (CD44^{+}CD62L^{-}), and memory (CD44^{+}CD62L^{+}) CD8^{+} T cells (**A** and **B**) and CD4^{+} T cells (**C** and **D**) in the spleens of indicated mice. Data are representative of ≥3 independent experiments with ≥3 mice per group. In dot plots, each symbol represents an individual mouse. Statistics were calculated using Student's t-test; *, $p \leq 0.05$; **, $p \leq 0.01$; ***, $p \leq 0.001$; ****, $p \leq 0.0001$; ns, not significant, $p > 0.05$.

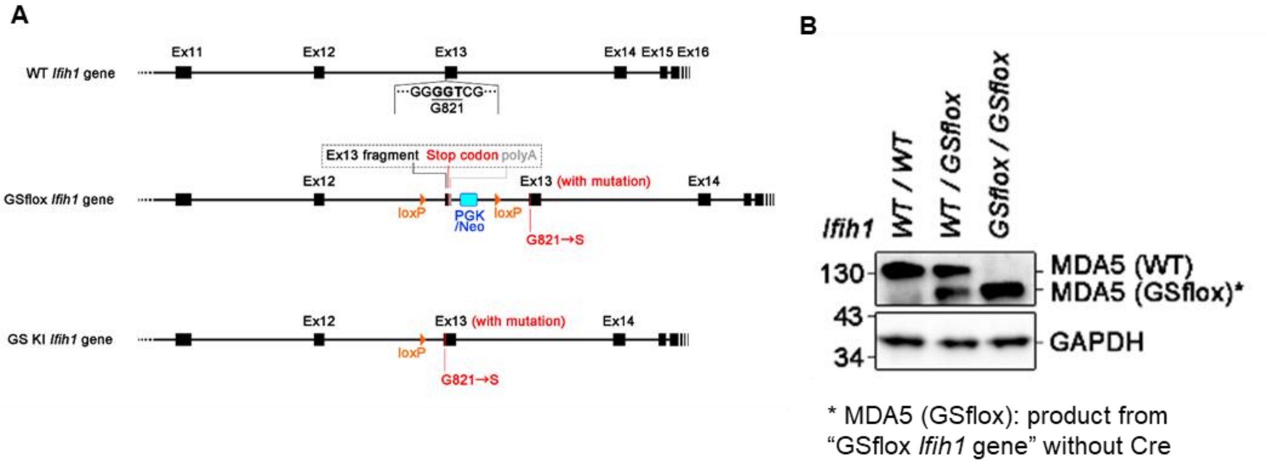

Fig. S5. Conditional MDA5 G821S expression system. (**A**) Schematic overview of the conditional MDA5 G821S (GS) expression system. Top: WT *Ifih1* gene with the indicated site of mutation (GGT) in exon 13, which encodes for glycine at position 821 (G821). Middle: GS flox *Ifih1* gene with a one-base substitution mutation (G to A, AGT), resulting in the replacement of glycine to serine (G821S). This mutation is preceded by a stop codon cassette flanked by loxP sites, resulting in the expression of a truncated MDA5 protein. Bottom: GS flox *Ifih1* gene with the stop codon deleted in the presence of Cre recombinase, allowing the expression of the MDA5 GS mutant protein. (**B**) Western blotting of MDA5 protein in the spleen of wild-type (WT/WT), heterozygous MDA5 GS (WT/GS flox), and homozygous MDA5 GS (GS flox/GS flox) mice without Cre recombinase expression.

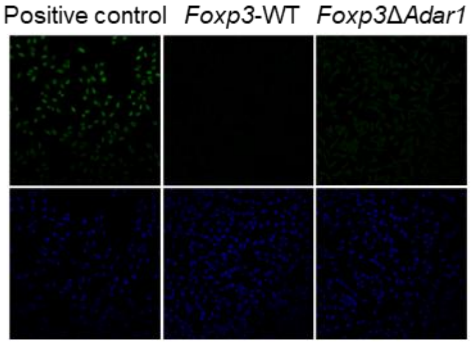

Fig. S6. Immunofluorescent staining for the detection of anti nuclear antibodies (ANA). Immunofluorescence staining of L929 cells using sera from *Foxp3*-WT (representative of 5 mice) and *Foxp3*$^{\Delta Adar1}$ mice (representative of 8 mice) (top green, ANA; bottom blue, DAPI).

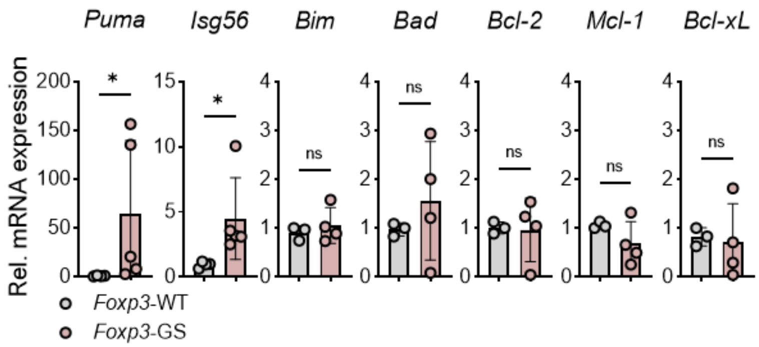

Fig. S7. Pro- and anti-apoptotic gene expression in Tregs from *Foxp3*-GS mice. Relative mRNA expression of indicated genes in $CD4^{+}YFP^{+}$ ($FOXP3^{+}$) Tregs sorted from spleens. In dot plots, each symbol represents an individual mouse. Data are representative of ≥3 independent experiments with ≥3 mice per group. Statistics were calculated using Student's t-test; *, $p \leq 0.05$; ns, not significant, $p > 0.05$.

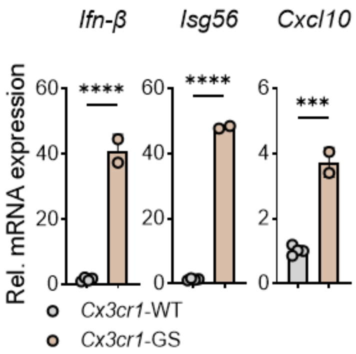

Fig. S8. ISG signature in *Cx3cr1*-GS mice. Relative mRNA expression of indicated genes in the spleens. Data is representative of 2 independent experiments with ≥2 mice per group. Each symbol represents an individual mouse. Statistics were calculated using Student's t-test; ***, $p \leq 0.001$; ****, $p \leq 0.0001$.

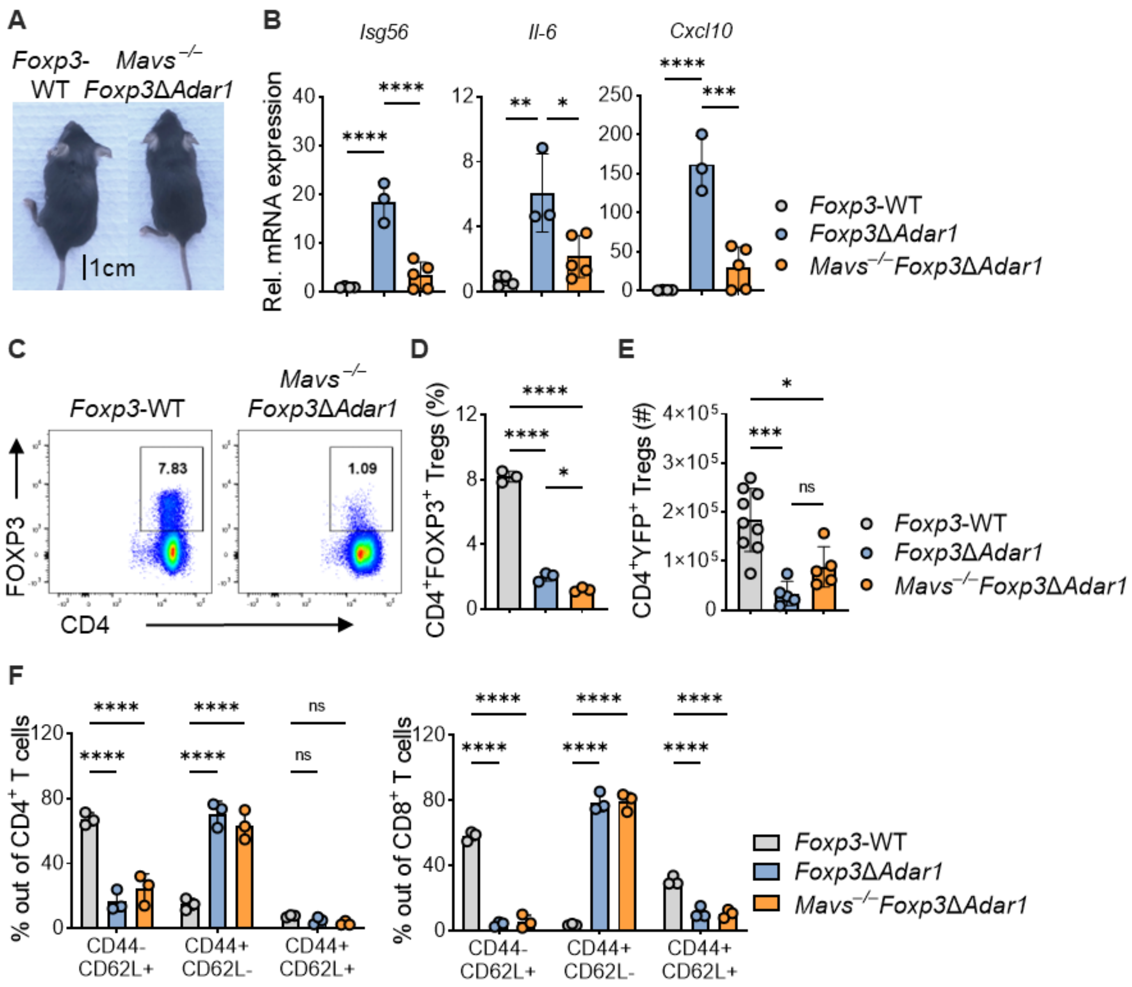

Fig. S9. Kidney inflammation, Treg loss and increased effector T cells in *Mavs*$^{-/-}$*Foxp3*$^{\Delta Adar1}$ mice. (**A**) Representative pictures of 3-weeks-old *Foxp3*-WT and *Mavs*$^{-/-}$*Foxp3*$^{\Delta Adar1}$ mice. (**B**) Relative mRNA expression of indicated genes in the kidneys. (**C-E**) Representative flow cytometry plots and summarized percentages (%) of CD4^{+}FOXP3^{+} Tregs in the inguinal lymph nodes (ILNs), and total numbers (#) in the spleens. (**F**) Summarized percentages of naive (CD44^{-}CD62L^{+}), effector (CD44^{+}CD62L^{-}), and memory (CD44^{+}CD62L^{+}) CD4^{+} and CD8^{+} T cells in the spleens of 3-weeks-old *Foxp3*-WT, *Foxp3*$^{\Delta Adar1}$, and *Mavs*$^{-/-}$ *Foxp3*$^{\Delta Adar1}$ mice. Data are representative of ≥3 independent experiments with ≥3 mice per group. In dot plots, each symbol represents an individual mouse. Statistics were calculated using one-way ANOVA; *, $p \leq 0.05$; **, $p \leq 0.01$; ***, $p \leq 0.001$; ****, $p \leq 0.0001$; ns, not significant, $p > 0.05$.

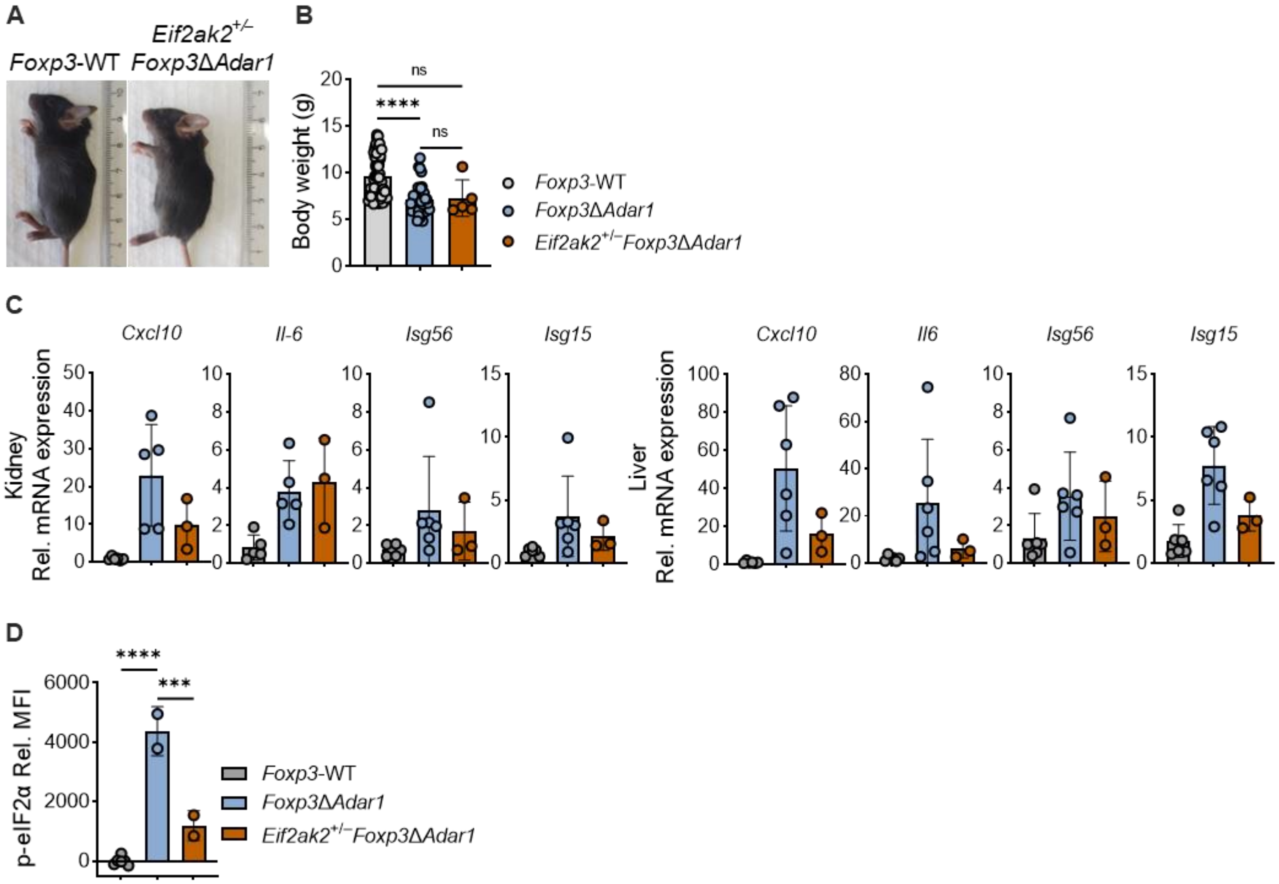

Fig. S10. Phenotypic analysis of *Eif2ak2*$^{+/-}$*Foxp3*$^{\Delta Adar1}$ mice. (**A**) Representative pictures of 3-weeks-old *Foxp3*-WT and *Eif2ak2*$^{+/-}$*Foxp3*$^{\Delta Adar1}$ mice. (**B**) Body weight measurements of 3-weeks-old mice, *Foxp3*-WT (n=44), *Foxp3*$^{\Delta Adar1}$ (n=36), and *Eif2ak2*$^{+/-}$*Foxp3*$^{\Delta Adar1}$ (n=4). (**C**) Relative mRNA expression of indicated genes in the kidneys and livers. (**D**) Phospho-eIF2α mean fluorescence intensity (MFI) values in CD4$^+$FOXP3$^+$ Tregs, relative to controls (*Foxp3*-WT). *Eif2ak2*$^{+/-}$*Foxp3*$^{\Delta Adar1}$ mice data is representative of 5 individual mice. Each symbol represents an individual mouse. Statistics were calculated using one-way ANOVA; ***, $p \leq 0.001$; ****, $p \leq 0.0001$; ns, not significant, $p > 0.05$.

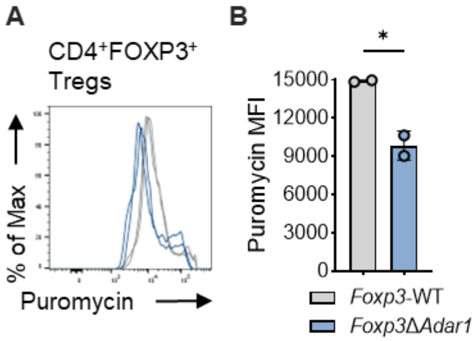

Fig. S11. Puromycin incorporation by Tregs. (**A**) Representative histograms and (**B**) MFI values of puromycin staining in $CD4^{+}FOXP3^{+}$ Tregs. $CD4^{+}$ T cells were enriched from splenocytes by negative selection, incubated with puromycin for 45 minutes, stained and analysed by flow cytometry. Data is representative of 3 individual experiments with ≥2 mice per group. Each symbol represents an individual mouse. Statistics were calculated using Student's t-test; *, $p \leq 0.05$.

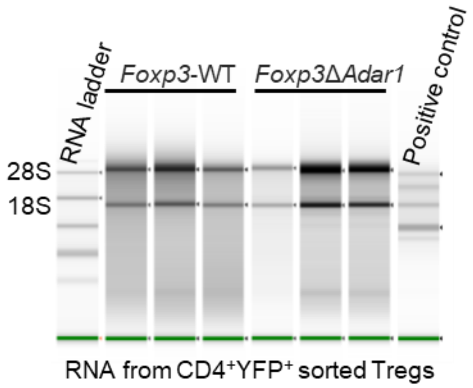

Fig. S12. Automated gel electrophoresis of RNA from *Foxp3*$^{\Delta Adar1}$ Tregs. RNA was isolated from CD4^{+}YFP^{+}(FOXP3^{+}) Tregs sorted from the spleens of *Foxp3*-WT and *Foxp3*$^{\Delta Adar1}$ mice. Each lane represents RNA from an individual mouse (in total, RNA from Tregs of 6 *Foxp3*$^{\Delta Adar1}$ has been checked). Positive control: RNA from A549 cells treated with Poly(I:C) for 6h.

Table S1.
Information on the patients with Aicardi–Goutières syndrome (AGS).

Patient number	Sex	Age (years)	Mutation	Treatment	Disease
1	M	4	*ADAR1*, p.Gly1007Arg, heterozygous de novo	Ruxolitinib	AGS, ISG signature
2	F	3	*ADAR1*, p.[Ala813Glnfs];[Pro193Ala]	No JAKi	AGS, ISG signature
3	M	8	*IFIH1*, p.Ile416Leu, heterozygous de novo	No JAKi	AGS, ISG signature
4	F	5	*ADAR1*, p.Gly1007Arg, heterozygous	No JAKi	AGS, ISG signature
5	F	14	*ADAR1*, p.Gly1007Arg, heterozygous	No JAKi	AGS, ISG signature
6	F	3	*IFIH1*, p.Arg779His, heterozygous	No JAKi	AGS, ISG signature
7	M	7	*ADAR1*, p.[Met1?];[Pro193Ala]	No JAKi	AGS, ISG signature, mild symptoms
8	F	3	*IFIH1*, c.2156C>T, p.Ala719Val, heterozygous de novo	Ruxolitinib, SCIg	AGS, ISG signature
9	M	4	*ADAR1*, p.Gly1007Arg, heterozygous	No JAKi	AGS, ISG signature
